# The Development of Novel Functional Corn Flakes Produced from Different Types of Maize (*Zea mays* L.)

**DOI:** 10.3390/foods12234257

**Published:** 2023-11-24

**Authors:** Milenko Košutić, Ivica Djalović, Jelena Filipović, Snežana Jakšić, Vladimir Filipović, Milica Nićetin, Biljana Lončar

**Affiliations:** 1 Institute of Food Technology, University of Novi Sad, Bulevar Cara Lazara 1, 21000 Novi Sad, Serbia; jelena.filipovic@fins.uns.ac.rs; 2 Institute of Field and Vegetable Crops, National Institute of the Republic of Serbia, Maxim Gorki 30, 21000 Novi Sad, Serbia; ivica.djalovic@ifvcns.ns.ac.rs (I.D.); snezana.jaksic@ifvcns.ns.ac.rs (S.J.); 3 Faculty of Technology Novi Sad, University of Novi Sad, Bulevar Cara Lazara 1, 21000 Novi Sad, Serbia; vladaf@uns.ac.rs (V.F.); milican@uns.ac.rs (M.N.); cbiljana@uns.ac.rs (B.L.)

**Keywords:** corn types, flake product, extrusion process, quality optimization, functional product

## Abstract

Cereal products, such as flakes and snack items, are frequently consumed as part of everyday diets, encompassing ready-to-eat breakfast cereals, flakes, and snacks. The utilization of extrusion technology is crucial in the manufacturing process of cereal-based flakes or snack products. When it comes to cereal-based flakes or snacks, different types of corn, such as white corn, yellow corn, red corn, and black corn, have garnered attention from scientists, consumers, and experts in the food industry. This paper investigates the simultaneous effects of different types of corn (white corn, yellow corn, red corn, and black corn) addition and different screw speeds (350, 500, 650 rpm) on the physical, technological, and functional properties of flake products. An increasing screw speed had a positive influence on the physical and technological characteristics of corn flakes, while different types of corn had a positive influence on the mineral composition and antioxidant properties. Black corn flour and a screw speed of 350 rpm positively influenced the physical and technological characteristics, mineral composition, and antioxidant properties of flake products, with a best total Z-score analysis of 0.59. Overall, the combination of Tukey’s HSD test and PCA enabled a comprehensive analysis of the observed corn products and allowed us to identify satiating and significant differences between attributes and create a classification of the samples based on those differences. Corn flakes from black corn flour on a screw speed of 350 rpm is a new product with good physical–technological and functional properties due to a higher level of antioxidant activity. The last three samples have a significantly higher percentage of free radical inhibition compared with the other samples according to TPC and TFC. This product has the potential to be found on the market as a new product with functional properties.

## 1. Introduction

Corn is one of the most diverse grain crops and a widely cultivated cereal globally. It has a rich history and plays a crucial role in global agriculture and food systems. Since the first historical report, maize seeds were described as having different colors such as white, red, and black [1]. The corn’s color is mainly due to the presence of a huge number of secondary metabolites, such as phenolic acids, carotenoids, and flavonoids [2]. Various corn types have been cultivated and consumed by different cultures around the world for centuries, each with its own unique color, taste, texture, and uses. Whether it is for human consumption, livestock feed, or ornamental purposes, corn is a truly versatile and diverse crop.

The development of novel colored genotypes aims to expand the availability and commercial production of pigmented maize varieties. These pigments are considered to be physiologically active components and/or health promoters since their role in promoting good health and reducing the risk of chronic diseases has been scientifically documented [3]. However, it is important to note that the specific levels and types of antioxidants and bioactive compounds can vary among different varieties of pigmented corn. Natural antioxidants are found in many types of grains and corn. Anthocyanin pigments from a wide variety of edible and ornamental black, blue, pink, purple, red, and white wheat, barley, corn, rice, and wild rice were identified and quantified to evaluate their potential as natural colorants or functional food ingredients [3]. As natural colorants, anthocyanins can be used to enhance the visual appeal of food products, replacing synthetic color additives. Additionally, their antioxidant properties make them attractive as functional food ingredients, as they can contribute to the health benefits associated with a diet that is rich in antioxidants.

Food enriched with bioactive component has become popular [4], and for that reason, colored corn, which is a member of the coarse grain family, has attracted extensive attention for its considerable amounts of anthocyanins and other bioactive components, which have positive functions in human health. Colored corn has a lot of bioactive components such as antioxidant, anti-inflammatory, anticancer, antidiabetic, and ocular health-enhancing agents [5,6,7].

Extrusion cooking is a high-temperature, high-pressure, short-time, low-cost processing technique performed worldwide that allows for the production and modification of various products [8]. The extruded food products’ quality is remarkably dependent on the feed material composition, barrel temperature profile, feed moisture content, screw speed, and other extrusion system parameters [9]. If the aim is to produce a quality product, extrusion is a complex multi-variable process requiring optimization of working parameters and careful control of the process [10]. 

Snacks are an important part of the diet. Snack foods have experienced an increase in popularity because their consumption provides the sense that the appetite is satisfied. In the late 1970s and early 1980s, manufacturers of snack foods and, to a lesser degree, ready-to-eat breakfast cereals and fast-food chains came under criticism for selling “empty calorie” foods. Many snack products are classified as junk food due to their low nutrient content, high preservative content, and the presence of other ingredients that are known to be harmful to consumer health [11]. Snack foods are generally produced by using cereal-based refined flour or starch, and many of them are relatively high in sugar, fat, and salt, hence being considered energy-dense but nutritionally poor foods [9]. Snack foods have become an important part of the diet for all age groups, especially children, for being cheap and fast to eat. Approximately 25% of children’s total daily caloric intake is through snack foods [12]. In recent years, with the growth of the market for healthy and sustainable diets, and with increased consumer awareness of diet, health, and the environment, there has been a change in the interests and habits of contemporary consumers within the field of food during last decades. People have become conscious of their health, which has raised their interest in the consumption of food that is nutrient-enriched as well as in functional foods. Consumers’ demand for access to high-value food products is increasing day by day [12]. The availability of nutritional information and health-related content on the internet has made it easier for consumers to educate themselves about food and choices. Consumers look for food articles that combine an interesting appearance, highly acceptable crunchy texture, and appealing flavor but with nutritive or functional ingredients [13]. The enrichment of food with the addition of functional ingredients is a promising strategy to enhance the health benefits of different foods [14]. 

By seizing the opportunity to develop innovative, healthy, and eco-friendly extruded snacks, one can tap into the growing market of health-conscious and environmentally aware consumers while contributing to a more sustainable food industry. 

The objective of this research is to optimize the production of flake products from different types of corn flour (white, yellow, red, and black) in order to test the effect of different mechanical energy inputs on the physical, technological, and functional properties of flake products.

## 2. Materials and Methods

### 2.1. Materials

In this study, four different types of corn (*Zea mays*) with different pigmentations were used (white corn = WC, yellow corn = YC, red corn = RC, and black corn = BC). Corn flour was obtained from the Institute of Field and Vegetable Crops in the year 2022, Novi Sad, Serbia. In Table 1, Table 2, Table 3 and Table 4, color, mineral characteristics, and biochemical characteristics of flour from different types of corn are presented. Different types of corn flour are characterized by moisture [15] and particle size [16] in Table 1. 

L-ascorbic acid, ferric chloride, gallic acid and 2,2-diphenyl-1-picrylhydrazyl (DPPH) were purchased from Sigma-Aldrich Chem (Steinheim, Germany). Folin-Ciolcateu reagent was provided by Fisher Scientific (Leicestershire, UK). 2,4,6-tripyridil-s-triazine (TPTZ) were acquired from Merck, Damstadt, Germany AlCl_3_, methanol and ethanol were purchased from Merck (Darmstadt, Germany).

### 2.2. Extrusion Process

**Figure 1:**
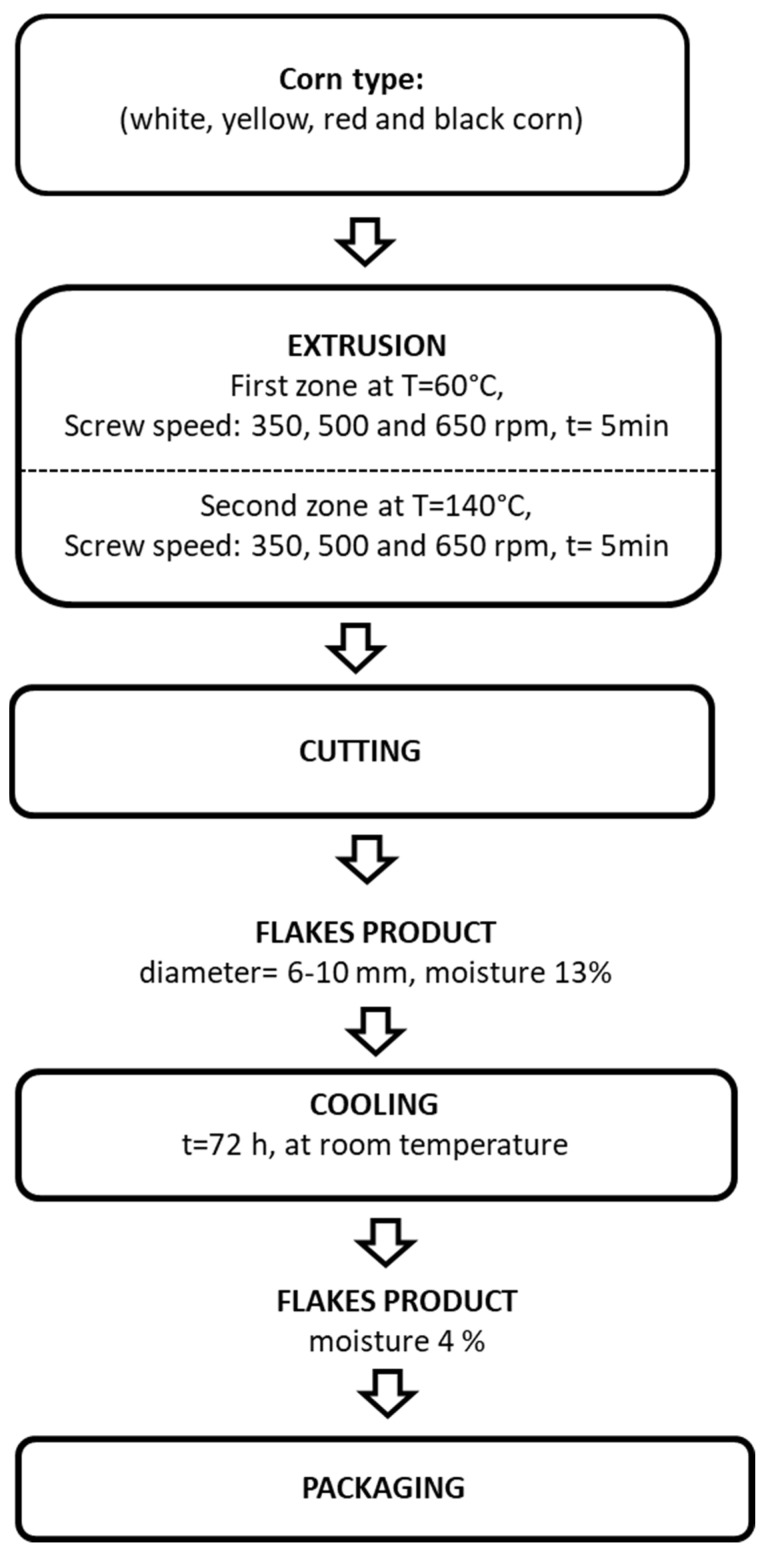
Schematic technological process of extrusion corn flakes.

**Figure 2:**
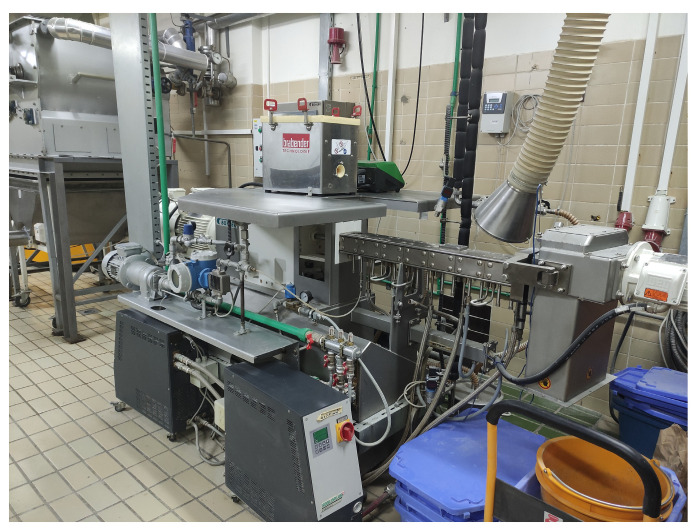
Picture of twin-screw rotating extruder Bühler BTSK 30/28D.

Table 4 shows the experimental design of different mechanical energy inputs and different corn types in the process of the corn flake production. Figure 1 shows a schematic illustration of the technological process of extrusion. Flake products were prepared using rotating twin-screw extruder Bühler BTSK 30/28D (Uzwil, Switzerland) (Figure 2) with 7 sections, and length/diameter ratio = 28:1. Screws fit together and rotate in the same direction. The total length of the extruder barrel was 880 mm. The extruder was equipped with two tempering tools for heating/cooling of the sections. The first tempering tool had tempering water temperature set at 60 °C for zones 2–4. For the second tool, water temperature was set at 140 °C for zones 6–7. A die plate that had one 4 mm diameter opening with cone inlet (total die opening was 12.56 mm^2^) was used. A screw configuration specially designed for production of directly expanded extruded products was used. A feed rate 25 kg/ha and screw speed (250, 500, 650 rpm) during extrusion were used. The extruder was equipped with a water pump that was used for the addition of water directly into the feeding sector of the barrel, enabling changes in the moisture content of the material. The rate flow of water was set according to the feed rate and starting moisture of the corn flour (Table 1) in order to achieve desired moisture content of 25% according to the experimental design. Sensors for die pressure and temperature were mounted at the material outlet. All extrusion data, including die temperature, pressure at the die, motor load, and specific mechanical energy, were read and downloaded directly from the PLC unit of the extruder. For achieving final length of the product, a cutter at the outlet of the material from the die of the extruder was fitted with six knives, with a rotational speed set at 450 rpm. Flake products were analyzed for their physical, technological, and functional properties 72 h after production.

### 2.3. Flake Characterization

#### 2.3.1. Bulk Density

Bulk density (BD) of flakes products was calculated as the ratio of flake mass and cylinder volume [17]. Six samples were used for each flake product to calculate the mean.

Bulk density = Weight of flakes (kg)/Cylinder volume (m^3^)

#### 2.3.2. Expansion Index 

Expansion index (EI) was calculated as the ratio of the flakes and die diameters according to Vallée [18]. Six samples were used for each flake product to calculate the mean. 

### 2.4. Texture Properties

The texture characteristics of flake products were determined using a texture analyzer TA-XT2 Texture Analyzer (Stable Micro System, Godalming, UK) according to modified method (dry cat food P35). Tests were performed by placing 3 flake products from each sample on a flat surface of the device and applying compression using a cylindrical stainless steel probe with 45 mm diameter (P45) at a load cell of 50 kg and a breaking force of 100 g. The setting parameters during the test were as pretest, test, and post-test speeds that were adjusted by 2.00, 1.00, and 10.00 mm s^−1^, respectively. The hardness of the flakes is defined as the force required to achieve the first fracture of the flakes, read as the maximum value from the obtained curve of force to the probe path dependence (N). Flake textural characteristic measurements were performed in six repetitions, for each flake batch in 12 samples, 72 h after production, at 25 °C.

### 2.5. Color Instrumental Analysis

Color parameters of flake samples were determined in six replications, 72 h after production, using Chroma meter (CR-400, Konica, Minolta, Tokyo, Japan) tri-stimulus colorimeter (contact surface diameter: 8 mm). Prior to sample measurements, calibration was performed using the white color standard. Color parameter results were presented according to CIElab color system, where coordinates are defined as follows: L*—brightness (from 0 (black) to 100 (white)), a*—greenness/redness (from −a* (green) to +a* (red)), b*—blueness/yellowness (from −b* (blue) to +b* (yellow)) [10,19].

### 2.6. Mineral Characteristics

Mineral element concentrations were determined via inductively coupled plasma atomic emission spectrometry (ICP-AES) [20,21].

### 2.7. Biochemical Characteristics

Total polyphenolics (TPs) and polyphenolic groups (Tflav-flavonoids, TT-tannins, Tpro-proanthocyanidins, Tant-anthocyanidins) and antioxidant tests were extracted with acidified methanolic solution (MeOH:H_2_O:CH_3_COOH, 140:50:10). All total polyphenolic groups were expressed as mg/g dw and determined via methods explained in detail in the following references: TP, TT and TPro by Makkar [22], TFlav by Pękal and Pyrzynska [23], Tant by Lee [24]. 

DPPH* scavenging assay was carried out according to the method reported by Chan et al. [25], using vitamin C as a reference antioxidant. Sample (0.5 g) was extracted with 25 mL of methanol by continuously shaking for 1 h at room temperature. Thereafter, the mixture was centrifuged at 3000 rpm for 10 min, and the supernatant (extract) was collected. A portion of 2 mL of DPPH* solution (5.9 mg/100 mL methanol) was mixed with varying dilutions of the extract, amounting to 1 mL. The mixture was incubated in the dark for 30 min at room temperature, after which the absorbance was read at 517 nm. Afterwards, the DPPH* scavenging ability of the extract was calculated and expressed in terms of SC50 (that is, the concentration of extract that scavenged DPPH* by 50%) [26].

### 2.8. Methods of Statistical Analysis

#### 2.8.1. Analysis of Variance

Descriptive statistical analyses for all the obtained technological parameters were expressed as the mean ± standard deviation (SD). The evaluation of one-way ANOVA analyses of the obtained results was performed using StatSoft Statistica 10.0^®^ software. Collected data were subjected to one-way analysis of variance (ANOVA) for the comparison of means, and significant differences were calculated according to post hoc Tukey’s HSD (honestly significant differences) test at *p* < 0.05 significance level, with 95% confidence.

#### 2.8.2. Correlation Analysis

R software v.4.0.3 (64-bit version) was used for calculation and plotting of the color plot diagram for all quality responses of different extruded corn flakes samples. The corrplot function was applied and the “circle” method and upper type enabled as a graphical tool for representation of the correlation between the tested samples’ responses.

#### 2.8.3. Principle Component Analysis

Principal component analysis (PCA) was used for the pattern recognition using assay descriptors in order to characterize and differentiate all analyzed samples and respective responses.

#### 2.8.4. Z-Score Analysis

The Z-Score analysis applies min–max normalization to the different flake products produced with different types of corn and at different screw speeds, transforming these response values from their original unit system to a new dimensionless unit system, where these different responses are comparable, and further mathematical calculations are applied [27,28]. 

The maximum value of normalized total Z-score presents the optimum value of all combined segment Z-scores, including all analyzed responses, indicating the optimum total quality of flake samples. The following equations show the calculation of individual segments’ Z-scores:

Technological characteristics of flake product segments’ Z-score:

(1) $$S_{1i}=\frac{\sum_{k=1}^{2}\left(\frac{xki-xk\;min}{xk\;min-xk\;max}\right)+\sum_{j=1}^{2}\left(1-\frac{xji-xj\;min}{xj\;min-xj\;max}\right)}{4}$$

where xk are expansion index and crispier work, and xj are bulk density and hardness.

Instrumental color of flake product segments’ Z-score:

(2) $$S2i=\frac{\sum_{l=1}^{2}\left(\frac{xli-xl\;min}{xl\;max-xl\;min}\right)+\sum_{m=1}^{1}\left(1-\frac{x\;mi-xm\;min}{xm\;\max-xm\;min}\right)}{3}$$

where xl are L and b, and xm is a.

Mineral compositions of flake product segments’ Z-score:

(3) $$S3i=\frac{\sum_{n=1}^{6}\left(\frac{xni-xn\;min}{xn\;max-xn\;min}\right)}{6}$$

where xn are N, Ca, Fe, Mg, Na, Zn.

Biochemical characteristics of flake product segments’ Z-score:

(4) $$S4i=\frac{\sum_{o=1}^{5}\left(\frac{xo\;i-xo\;min}{xo\;\max-xo\;min}\right)}{5}$$

where xo are TPC, TFC, TAC, DPPH test, and DPPH test ASCA.

Total quality characteristics’ Z-score:

(5) Si=0.30·S1i+0.15·S2i+0.25·S3i+0.30·S4i

where flake products’ functional characteristics’ Z-score values represent 55%, while technological quality characteristics’ Z-score values represent 45% of the total Z-score or total quality.

max [Si]→optimum.

Z-score calculation was performed using Microsoft Excel ver. 2016.

## 3. Results and Discussion

### 3.1. Physical and Technological Characteristics of Flake Products

In every technological process, it is very important to accurately define the production parameters of the process to achieve a good and consistent product quality while minimizing the loss of the nutritional and functional properties of the raw materials [10,29]. Through the process of extrusion using mechanical, thermal, diffusional, and chemical operations, the properties of the initial raw materials have been altered, primarily due to the characteristics of the extruder and the extrusion conditions (temperature, pressure, and shear stress according to Košutić [10,29]).

The physical and technological characteristics of flake products from different types of corn and screw speeds are given in Table 5. For flake products, one of the important parameters is bulk density (BD) because it defines volume, shape, and size of products [30]. The ANOVA test shows that the increase in screw speed (from 350 to 600 rpm) influences to a statistically significant degree the decrease in BD in the same type of corn (between samples: 1–3, 4–6, 7–9, 10–12). The BD depends on the type of corn. There are statistically significant differences between samples at the same screw speed and different types of corn (compered samples 1, 4, 7, 10, samples 2, 5, 8, 11, samples 3, 6, 9, 12). The highest values of BD are found in the samples from WC (sample 1–3), followed by YC (sample 4–6), and RC (sample 7–9), and the smallest values are registered in extruded products from BC (10–12). An enhancement in screw speed resulted in flake products with a lower BD. According to Alam [31] and Bokić [30], a high screw speed increased the process energy input; consequently, it accelerated a high sudden pressure drop at the die, contributing to less dense extruded products.

**Figure 3:**
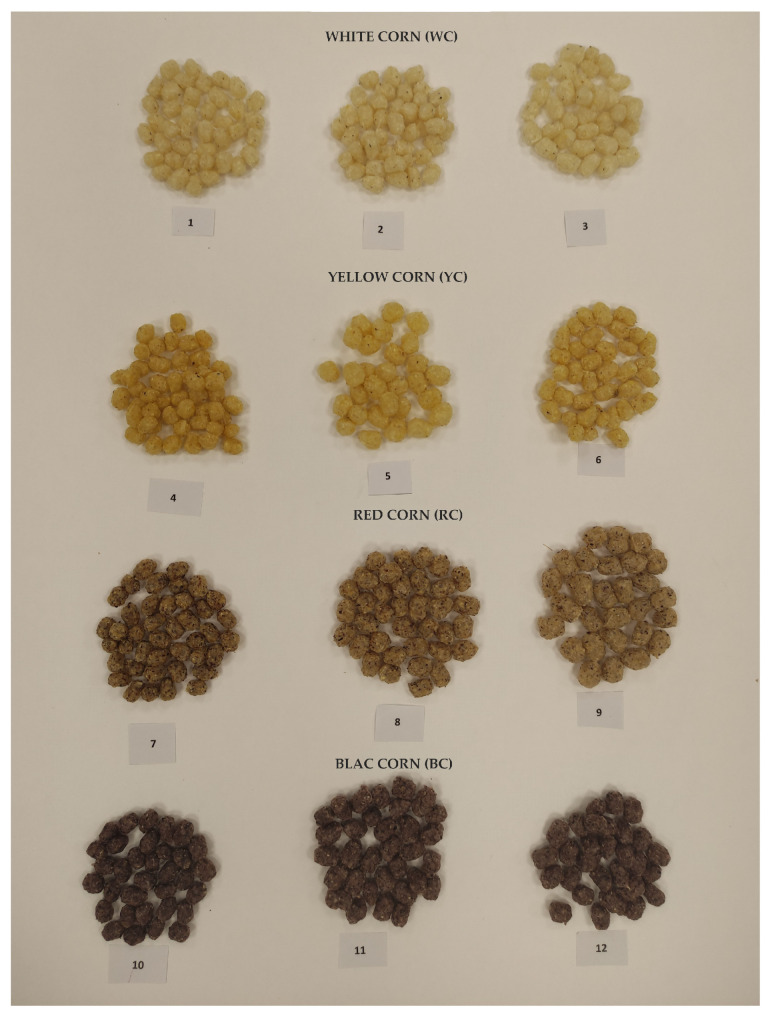
The images of extruded product produced from different types of corn and screw speed degrees of The samples 1, 4, 7 and 10 = 350 rpm; 2, 5, 8 and 11 = 500 rpm; 3, 6, 9 and 12 = 650 rpm.

The screw speed plays a crucial role in achieving the desired expansion of the final product during the extrusion process [32]. The mechanical energy and type of corn influence the value of the expansion index (EI). The EI is directly related to screw speed. An enhancement of the screw speed contributed to an increase in the EI of the obtained extruded products (Figure 3 and Table 5). The EI of samples 7–9 and samples 10–12 significantly increased (*p* < 0.05) with an increasing screw speed (350 to 650 rpw). This trend may be explained by the gradual increase in shear forces inside the extruder barrel that is responsible for the material’s macromolecular degradation, resulting in a uniform dough, which consequently obtains better expansion properties [32]. The highest BD was recorded for sample 1 (26.77 kg/m^3^), which had the lowest EI (1.90), while sample 12 has the lowest BD (7.70 kg/m^3^) and the highest EI (3.3). For the process of extrusion, high values of the EI are desirable, and low values of the BD are preferable. These produce characteristics depend on the extrusion process parameters [33].

The specificity of the flake product structure results from variations in the mechanical energy, accompanied by changes in textural characteristics, defined by the following parameters: hardness and crispiness, as shown in Table 5. The textural characteristics of the flake products are one of the most critical factors affecting consumer acceptability [10,29]. 

Tukey’s test, at a *p* < 0.05 level of significance, showed that an increasing screw speed (from 350 to 600 rpm) resulted in a statistically significant decrease in hardness in the same type of corn (samples 1–3, samples 4–6, samples 7–9, samples 10–12). This phenomenon is likely attributable to the rapid screw speed, which generated a force that propelled the material towards the outlet. As a result, the extruders were able to expand more effectively, resulting in smoother end products. The same influence was reported by Tsokolar-Tsikopoulos [34] and Bokić [30]. These data show that the highest degree of hardness was achieved at a higher screw speed. The hardness of flake products also depends on the type of corn. Samples 1–3 and 4–6 from WC and YC are significantly harder compared with samples 7–9 and 10–12, which are made of RC and BC. There are statistically significant differences between samples prepared at the same screw speed and between different types of corn (we compared samples 1, 4, 7, and 10, samples 2, 5, 8, and 11, and samples 3, 6, 9, and 12). It is noted that the hardness (HAR) of the flake products displayed an inverse relationship with the expansion index EI. The same trends are reported by Kesre and Masatcioglu [9], who investigated extruded corn supplemented with red lentil bran. This can be explained by the fact that a more expanded extrusion product had longer but thinner cell walls. This led to an ease of crushing the product under compression [35]. 

In general, an increasing screw speed caused decreasing crispiness values in all samples (1–3, 4–6, 7–9, 10–12). These tendencies are found to be statistically significant at *p* > 0.05. It is also noticed that for different types of corn, there were statistically significant differences in crispiness between samples 1, 4, 7, 10; samples 2, 5, 8, 11; and samples 3, 6, 9, 12. The highest values of this parameter have samples from WC (12.98–5.87), and the lowest value from BC (4.41–2.04). The textural parameters of hardness and crispiness exhibit the same change trends as the bulk density at the same screw speed. These results were in agreement with the research by Anton [36], who concluded that textural properties were highly dependent on the expansion index, i.e., that extruded products with a higher expansion index had lower hardness.

The images of extruded products from different types of corn and screw speed degrees of are presented in Figure 3. These images are provided for visual comparison and provide a preliminary insight into the physical quality properties of the extrusion products such as the expansion index and color values.

The content of pigments is characteristic of the corn type, which affects the products’ color and the overall quality. These facts about product color are important parameters as a significant indicator of the extruded product quality, which could affect their acceptability by consumers [10,29].

Table 1 presents the color of different types of corn flour (white, yellow, red, and black corn flour). It can be observed that the brightness (L) of the flake products increased after the extrusion process in samples 1–3, 7–6, and 7–9 (Table 6) compared with the raw material (Table 1). According to Anton [36], this fact was attributable to the incorporation of air into the structure of the extrudate. In the case of samples of black corn (10–12), the brightness decreased in comparison to raw material (Table 1), and actually, the flake products (10–12) are darker (Table 6). The raw material of WC and YC flour (Table 1) is characterized by its redness value (a*), but after the extrusion process (sample 1–3 and 4–6), the redness value is overtaken by the greenness value (a*) (Table 6). In samples 7–9 and 10–12, the share of red color after the extrusion process has increased in relation to the raw material from RC and BC (3.63 and 1.36) (Table 1). Changes in the sheer green and red colors in the raw material and the finished product are due to different pigments in the type of corn. The share of yellow (b) color is increasing after extrusion in sample 1–3 and samples 4–6 (Table 6) compared with the raw material (Table 1) (WC, YC), while this parameter is inversely correlated to the extrusion process in samples 7–9 and 10–12 of RC and BC (Table 6) compared with the raw material in RC and BC (Table 1), respectively.

Table 6 shows the results of the instrumental color measurements of flake products from different types of corn through the parameters of brightness (L) and share of individual tones (a and b). Statistical analysis at a significance level of *p* < 0.05 indicated that the change in screw speed (350, 500, 650 rpm) has no statistically significant effect on the observed color parameters (L, a, b) in individual corn types (1–3, 4–6, 7–9, 10–12). The color of flake products depends on the type of corn (Figure 3 and Table 6). 

There is a statistically significant difference in the color parameters (L, a, b) between the types corn at the same screw speed (samples 1, 4, 7, 10; samples 2, 5, 8, 11; samples 3, 6, 9, 12). The highest brightness (L*) values are found in flake products from WC (1–3), then YC (4–6), and then RC (7–9), and the darkest products are the flake products from BC (10–12). Flake products from BC are 42% darker than flake products from WC; these results are in accordance with Yang and Zhai [37]. The share of green tone (a) was the highest in WC samples 1–3 (−0.56 to −0.68) and slightly smaller in the sample of YC (−0.99 to −0.72), while in samples 7–9, a red tone is dominating, while the highest share of redness is found in samples from BC (8.92 to 9.59). 

In all 12 samples, yellow tones are dominant. The highest share of yellow color (b) is found in flake products from YC (25.06–25.19), followed by RC and WC, and the lowest value of the share of yellow tone is registered in samples from BC (8.40–4.49).

### 3.2. Functional Characteristics of Flake Products

The mineral content of the starting raw materials depends on the type of corn, which is presented in Table 2. Daily requirements of minerals are small, especially in comparison to macronutrients, but they are essential for the normal functioning of the body. If minerals are not consumed in sufficient quantities, it can cause disturbances in the human metabolism and sometimes lead to more serious illnesses. For this reason, it is desirable to consume food that is rich in minerals [36].

In this paper, the mineral composition of flake products made from different corn types was also investigated (Table 7). The mineral content depended on the corn type, while different screw speeds had no statistically significant influence on the mineral content within the group of the same type of corn flake samples. Each mineral element has an important function in the human body, e.g., nitrogen (N) content is important in food because it is a component of proteins and essential amino acids [38]. The nitrogen content depends on the corn type, with the lowest content recorded in the flake product made from yellow corn, sample 4 (1.358), and the highest in the flake product made from black corn, sample 10 (1.697). The flake products made from white and red corn have approximately the same nitrogen content. 

Sulfur (S) is also a structural element of proteins and amino acids, which is suppling the human body through food intake [38]. The percentage of sulfur is higher in flake products made from yellow and white corn: 0.455, 0.402, respectively, at the lowest screw speed (350 rpm). 

The boron (B) content in flake products depends on the corn type. The highest B content is observed in sample 1 (7.305), while the lowest is in sample 12 (4.845). It is observed that in the samples of flake products made from white corn (1–3) and black corn (10–12), the boron content was similar. 

Calcium (Ca) is an essential mineral element that plays a crucial role in the composition of bones in the human body, and its deficiency can lead to various disorders [38]. The highest Ca content is detected in the flake products made from white corn (1–3), followed by yellow corn (4–6), while a significantly lower Ca content is observed in the samples made from red (7–9) and black (10–12) corn. 

The availability of copper (Cu) ingested through food in the human body is often hindered by liver damage. Cu is one of the constituents of human immune cells [38]. The Cu content in flake products depends on the corn type and the input of mechanical energy. The ANOVA test had shown a statistically significant increase in the Cu content in the flake products made from white corn (1–3) and yellow corn (4–6), parallel to an increasing screw speed (form 350 to 650 rpm). A similar trend is also observed in the flake products made from red and black corn, while deviations are noted in samples 8 and 11.

Iron (Fe) is a mineral element that needs to be supplied through food, as it contributes to the blood circulation in the human body [38]. The highest Fe content is observed in flake products made from white corn (1–3), followed by yellow corn (4–6), while a significantly lower Fe content is found in the samples made from red (7–9) and black (10–12) corn. 

Magnesium (Mg) is an essential element for the functioning of enzymes in the body. It is particularly important to ensure an adequate intake of Mg in cases of higher physical exertion, where muscle-related issues can arise [38]. The Mg content in all 12 samples was relatively consistent. The Mg content ranges from 886.9 to 1001. The highest levels of Mg were recorded in sample 1, while the lowest values were found in sample 4.

Manganese (Mn) is important for preventing allergic and asthmatic symptoms in the human body [38]. The flake products made from white corn (samples 1–3) had a statistically significantly higher Mn content compared with other samples at the same screw speed. Samples from yellow (4–6), red (7–9), and black corn (10–12) have approximately the same values of Mn content, with no statistically significant differences. 

Sodium (Na) plays a vital role in the human body as an essential component of electrolytes [32]. The content of Na in all 12 samples is relatively constant and ranges from 31.37 to 35.92. The highest levels of Na content are observed in sample 1, while the lowest value is found in sample 6. A similar trend is experienced as with Mg, where the highest levels of Na and Mg were found in samples from white corn (1–3), while the lowest levels were found in samples from yellow corn (4–6).

Phosphorus (P) is an important mineral element in the human metabolism and is found in various foods [38]. There is no statistically significant difference in the phosphorus content among the 12 samples of flake products. However, some trends can be observed. The lowest phosphorus content is registered in samples from yellow corn (4–6), followed by white corn (1–3) and red corn (7–9), while the highest phosphorus content is found in samples from black corn (10–12).

Zn (zinc) is a crucial element in the human body, and its deficiency can lead to significant metabolic changes. The zinc content was the lowest in sample 1 and highest in sample 10.

The data on the content total phenols, flavonoids, anthocyanins, and DPPH tests of the 12 samples of extruded flake products from different types of corn at different levels of applied mechanical energy are presented in Table 8. In the technology process of food extrusion, the aim is to preserve the natural antioxidant potential of the raw materials as much as possible [36]. 

Comparing the biochemical properties of the raw materials of different corn flour types (Table 3) and the biochemical properties of the flake products (Table 8), it can be observed that the technological extrusion process had a significant influence on the measured parameters. In Table 8, the data show that the total phenolic contents after the extrusion process are lower in all samples (1–12), the contents of total flavonoids are higher in all samples (1–12), and anthocyanins are higher in samples 7–12 compared with the initial raw materials (Table 3). After the extrusion process, the parameters of DPPH test% and DPPH test ASCA decreased in flake products made from WC (1–3), YC (4–6), and RC (7–9) compared with the initial raw materials (Table 3). This is in accordance with Košutić [10]. However, there is an increase in these parameters for flake products made from BC (10–12). 

The results of the analysis of the total phenol content showed that the type of corn lead to a statistically significant difference in these parameters. The highest content of total phenols was recorded in samples 10–12, i.e., black corn, followed by flake products from red corn (7–9), while flake products from yellow (4–6) and white corn (1–3) have the same content of total phenols, i.e., there is no statistically significant difference in this parameter. Concerning the same type of corn, changing the mechanical energy input did not have an impact on the observed parameters. 

There was a slight increase in the total flavonoid content in the samples of flake products made from white corn (1–3), yellow corn (4–6), and red corn (7–9), which was parallel to increasing the screw speed (350 to 650) (Table 8). However, in sample 11 made from black corn, the highest content of total flavonoids is detected. There is a statistically significant difference in the total flavonoid content between different corn types (1, 4, 7, 10; 2, 5, 8, 10; and 3, 6, 9, 12) at the same screw speed. In Table 8, it can be noted that darker corn types have a higher flavonoid content than lighter types of corn. The highest content of flavonoid is observed in the flake products made from black corn (10–12) (0.72–0.83), while the lowest flavonoid content is found in the flake products made from white corn (1–3) (0.41–0.44).

The results on the content of total anthocyanins in the 12 samples of flake products indicate the trend of the impact of corn type on the composition of flakes products (Table 8). The results on the content of total anthocyanins show that these are not detected in flake products from white and yellow types of corn (samples 1–3 and 4–6). Total anthocyanins are recorded in samples from red corn (ranging from 0.02 to 0.04), while a statistically significant increase was recorded in samples from black corn (0.44, 0.37, 0.33). The ANOVA test shows that with an increase in the screw speed (from 350 to 600), the content of anthocyanins significantly decreases in black corn (samples 10–12). These data indicate that the main source of anthocyanins in flake products is black corn. This product is rich in anthocyanins, a group of phenolic compounds. Anthocyanins are affecting the color of food, e.g., they cause a darker color of the product (Table 6), but also, they are marked as a healthy component of food. Similar findings are reported by Yang and Zhai [37].

The results of the antioxidant analysis of the 12 samples of flake products measured by the DPPH test % indicated a statistically significant difference in the impact of mechanical energy and corn type. Increasing the mechanical energy, the antioxidant potential decreased in flake products from white (1–3) and black corn (10–12). However, the opposite effect was recorded in flake products from yellow and red corn, where the antioxidant potential increases with an increase in the screw speed. A statistically significant difference in the DPPH test is recorded for different types of corn (sample 1–3, 4–6, 7–9, 10–2). This parameter depends on the type and content of pigments in the corn.

The antioxidant activity measured via the DPPH-ASCA method in flake products was significantly influenced by corn type, while the input of mechanical energy had no significant impact on this parameter [39]. The highest antioxidant activity measured via the DPPH-ASCA method in flake products was found in black corn (10–12), followed by red corn (7–9), and then yellow corn (4–6), while white corn (1–3) had the lowest value of this parameter. 

### 3.3. Results of the Statistical Analysis

#### 3.3.1. Results of the Correlation Analysis

**Figure 4:**
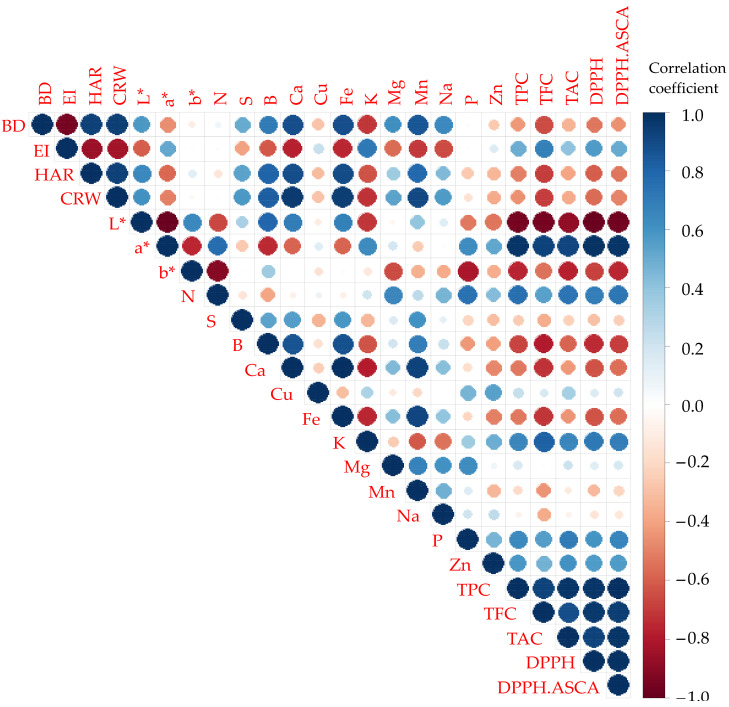
Color correlation diagram between all 24 tested responses of extruded flakes. Blue for positive and red for negative correlation; smaller size represents correlation value closer to 0, while bigger size represents values closer to 1.

A color correlation diagram between all 32 responses of the technological and instrumental color characteristics and the mineral and biochemical composition of the tested extruded flakes is shown in Figure 4.

A color correlation diagram is often used in determining the correlation between different technological and nutritive responses in cereal products [28].

Correlation coefficients’ values between any two responses are presented by different colors and sizes (blue for positive and red for negative correlation; a smaller size represents a correlation value closer to 0, while a bigger size represents values closer to 1).

The results of the correlation analysis showed a high level of negative correlation between the BD and EI and the EI and some minerals (Ca, Fe, Mn, and Na). There is also a negative correlation between the BD characteristics and a positive correlation between the EI characteristics of the groups of biochemical properties. The correlation coefficients between these parameters range from −0.34 to −0.66, indicating that corn type influences the flake samples’ biochemical properties more than extrusion parameters do.

A strong negative correlation is noticed between brightness and biochemical composition, and also, strong positive correlations between redness and the biochemical composition of the extruded flakes are shown, confirming already stated results and discussions presented for each individual response.

#### 3.3.2. Results of the PCA

PCA was used to analyze the structural correlation among different experimental responses and different tested extruded flake samples, which provides complementary information [40].

**Figure 5:**
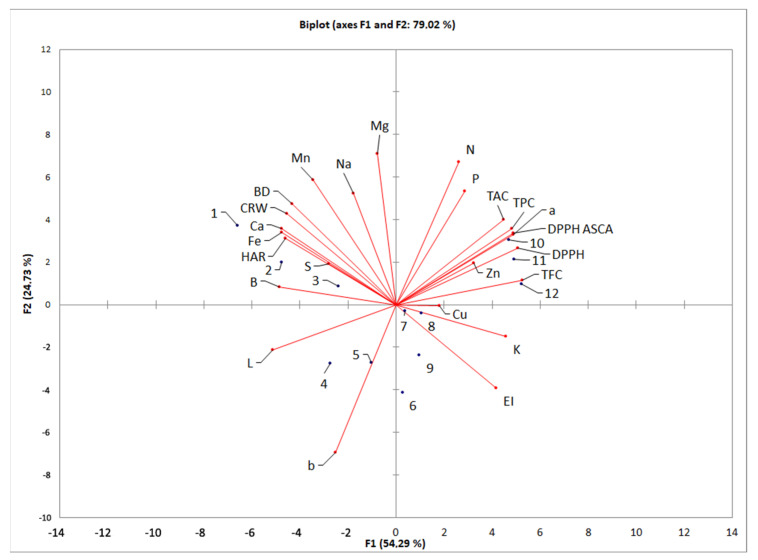
PCA of tested extruded flakes from different types of corn.

Figure 5 shows the PCA results, in which a scatter plot of samples was produced in an effort to produce a trend visualization of the data and discriminating the efficiency of the used descriptors. The first two principal components from the data matrix of PCA are presented: the first principal component at the x-axis and the second at the y-axis.

A neat separation of the twelve tested extruded flake samples according to different technological and functional quality responses can be seen from the presented scatter plot. The samples’ location in the figure was influenced by the corn type, distinguishing them from the negative to positive values of the first principal component (along the x-axis) and the screw speed, differentiating samples along the second principal component (along the y-axis).

From the PCA results it can be seen that extruded flake samples made with white corn flour were characterized by high values of all technological responses and brightness and low values of biochemical responses.

Extruded flake samples made with yellow corn flour were characterized by lower values of technological, mineral, and biochemical responses, while samples made with red corn flour were characterized by median values of all tested quality parameters. Extruded flake samples made with black corn flour were characterized by the highest values of biochemical and lowest values of technological responses.

The results of quality testing showed that the first two principal components accounted for 79.02% of the total variance; hence, it can be considered sufficient for data representation.

The responses that contributed the most to the first principal component were all biochemical responses, L and a of color responses, HAR and CRW of the technological responses, and B, Ca, Fe, and K of mineral matter responses, while the most significant responses to the second principal component were b, N, Mg, and MN.

#### 3.3.3. Results of the Z-Score Analysis

According to Košutić [10,29], Z-score analysis is often used in the optimization of raw material and flake products’ technological quality. Z-score analysis was applied to identify the optimal flake sample made from different types of corn at different screw speeds, focusing on the aspect of the 18 investigated technological and functional characteristics (Table 9). The presented results showed segment Z-scores S1, S2, S3, and S4, which correspond to the Z-score results of technological quality, instrumental color characteristics, selected mineral matter, and biochemical characteristics, respectively.

The Z-score values for technological characteristics, color, mineral compositions, and biochemical characteristics depend on the corn type and screw speeds. Higher values of segment Z-score S1 (0.51–0.75) and S4 (>0.82) were recorded for samples 10–12 from black corn, while the values of segment Z-score S2 were the best in samples from yellow corn (4–6), and segment Z-score S3 was the best in white corn samples (1–3). The total Z-score values mathematically combine all segment Z-scores and indicate the optimal combination of all 18 technological and functional characteristics. The maximum total Z score value (0.59) was achieved for sample 10 made from black corn and screw speed 350 rpm. Sample 10 had a good segment Z-score for technological, mineral, and biochemical composition, which influenced the total score. The minimum total score was registered in sample 4, which was obtained from yellow-type corn with minimal screw speed (350 rpm).

## 4. Conclusions

It can be concluded that different types of corn and screw speeds of the extruder affected the physical, technological, mineral, and biochemical characteristics of the flake products. Increasing the screw speed had a significant influence on the physical and technological characteristics of flake products from different types of corn: a decreased bulk density and hardness and an increased expansion index. Different types of corn showed a positive influence on the flake products’ color, mineral composition, and content of phenols, flavonoids, anthocyanins, and antioxidative activity compared with other samples.

The correlation analysis and PCA results confirmed the positive effect of red and black corn flour on functional characteristics, while the technological quality parameters were mostly negatively affected.

Flake products from black corn and an extrusion process at a screw speed of 350 rpm (sample 10) achieved the best total Z score value of 0.59 concerning technological and color characteristics, mineral composition, and biochemical properties combined.

The findings from this study can provide a novel functional product with good technological and biochemical characteristics, intended for consumers that are conscious about their health and diet. In future research, the plan is to expand this research with sensory analysis and consumer surveys.

## Figures and Tables

**Table 1:** Some characteristics of corn samples.

Corn Type				
	WC	YC	RC	BC
Moisture (%)	14.3	13.6	12.7	12.4
Particle size				
>350 µm	82.1	81.5	83.5	84.1
250–350 µm	9.5	8.7	8.6	7.0
150–250 µm	3.7	4.9	3.5	3.1
<150 µm	4.7	4.9	4.4	5.8
Color characteristics				
L	82.52	77.93	64.06	61.75
a	0.71	2.38	3.63	1.36
b	12.86	24.56	19.24	15.61

L—brightness (black/white), a—greenness/redness, b—blueness/yellowness.

**Table 2:** Mineral characteristics of flour from different types of corns.

Corn Type				
	WC	YC	RC	BC
N (%)	1.671	1.435	1.484	1.677
S (%)	0.248	0.244	0.253	0.250
B (%)	4.552	5.209	4.664	4.63
Ca (mg/kg)	701.50	687.20	699.30	677.70
Cu (mg/kg)	13.72	6.652	6.388	13.93
Fe (mg/kg)	1289	59.16	42.65	40.50
K (mg/kg)	2660	2720	2486	2822
Mg (mg/kg)	984.20	853.8	845.1	953
Mn (mg/kg)	73.11	6.932	6.016	7.712
Na (mg/kg)	99.78	112.80	113.00	112.00
P (mg/kg)	2566.3	2231.30	2130.0	2528.3
Zn (mg/kg)	29.78	20.72	19.54	26.20

N—nitrogen, S—sulfur, B—boron, Ca—calcium, Cu—copper, Fe—iron, Mg—magnesium, Mn—manganese, Na—sodium, P—phosphorus, Zn—zinc.

**Table 3:** Biochemical properties of flour from different types of corn.

Corn Type				
	WC	YC	RC	BC
TPC (mg/g d.m)	1.49	1.58	1.86	2.66
TFC (mg/g d.m)	0.03	0.07	0.11	0.35
TAC (mg/g d.m)	nd *	nd	0.0125	0.2705
DPPH-test%	60.09	69.23	84.75	80.94
DPPH-testug&g ASCA	2.66	3.21	4.41	4.08

TPC—total phenolic content, TFC—total flavonoids content, TAC—total anthocyanins content, DPPH-(2,2-Diphenyl-1-picrylhydrazyl), free radicals scavenging activity, antioxidant test, DPPH test. ASCA: The antioxidative activity of AOS was measured using the 2,2-diphenyl-1-picrylhydrazyl (DPPH) radical scavenging assay and the beta-carotene-linoleate assay systems and was compared with those of butylated hydroxytoluene and ascorbic acid (AscA). * nd—not detected.

**Table 4:** Experimental plan for producing flake products from different types of corn.

Sample	Type of Corn	Screw Speed (rpm)
1	White corn (WC)	350
2	White corn (WC)	500
3	White corn (WC)	650
4	Yellow corn (YC)	350
5	Yellow corn (YC)	500
6	Yellow corn (YC)	650
7	Red corn (RC)	350
8	Red corn (RC)	500
9	Red corn (RC)	650
10	Black corn (BC)	350
11	Black corn (BC)	500
12	Black corn (BC)	650

**Table 5:** Technological characteristics of flake products from different types of corn.

Sample	BD (kg/m^3^)	EI	HAR (N)	CRW (Nmm)
1	26.77 ± 0.21 ^g^	1.90 ± 0.08 ^a^	101.31 ± 18.97 ^e^	12.98 ± 2.73 ^f^
2	23.77 ± 0.434 ^f^	1.91 ± 0.06 ^a^	89.89 ± 14.4 ^de^	8.87 ± 0.91 ^e^
3	18.84 ± 0.25 ^e^	2.01 ± 0.05 ^a^	56.07 ± 20.42 ^a^	5.87 ± 0.81 ^d^
4	16.26 ± 0.21 ^c^	2.39 ± 0.07 ^a^	77.43 ± 17.18 ^cd^	5.66 ± 0.68 ^cd^
5	12.78 ± 0.28 ^b^	2.62 ± 0.14 ^bc^	59.63 ± 14.09 ^ac^	3.74 ± 0.55 ^abc^
6	7.73 ± 0.27 ^a^	3.14 ± 0.12 ^a^	39.92 ± 6.35 ^ab^	2.40 ± 0.35 ^a^
7	15.84 ± 0.74 ^c^	2.40 ± 0.09 ^a^	54.90 ± 12.48 ^a^	4.55 ± 0.87 ^bcd^
8	12.77 ± 0.28 ^b^	2.54 ± 0.07 ^b^	44.37 ± 9.23 ^ab^	2.97 ± 0.58 ^ab^
9	7.72 ± 0.14 ^a^	3.10 ± 0.13 ^c^	31.49 ± 6.81 ^b^	2.27 ± 0.29 ^a^
10	13.90 ± 0.36 ^d^	2.59 ± 0.10 ^a^	53.79 ± 11.30 ^a^	4.41 ± 0.46 ^bcd^
11	12.28 ± 0.19 ^b^	2.76 ± 0.11 ^b^	43.56 ± 8.40 ^ab^	2.27 ± 0.97 ^a^
12	7.70 ± 0.27 ^a^	3.30 ± 0.06 ^d^	30.45 ± 6.84 ^b^	2.04 ± 1.09 ^a^

Results represent average value (*n* = 6) ± standard deviation. Different letters in superscript in the same table column indicate a statistically significant difference between values at a level of significance of *p* < 0.05 (based on post hoc Tukey HSD test). BD—bulk density, EI—expansion index, HAR—hardness, CRW—crispier work (Nmm).

**Table 6:** Color characteristics of flake products from different types of corn.

Sample	L	a	b
1	87.21 ± 0.16 ^c^	−0.56 ± 0.09 ^a^	14.64 ± 0.14 ^b^
2	87.21 ± 0.13 ^c^	−0.58 ± 0.04 ^a^	14.57 ± 0.07 ^b^
3	87.33 ± 0.29 ^c^	−0.68 ± 0.10 ^a^	14.39 ± 0.13 ^b^
4	81.68 ± 0.33 ^b^	−0.77 ± 0.14 ^ab^	25.06 ± 0.36 ^d^
5	81.73 ± 0.41 ^b^	−0.99 ± 0.38 ^b^	25.13 ± 0.31 ^d^
6	81.40 ± 0.06 ^b^	−0.72 ± 0.05 ^ab^	25.19 ± 0.23 ^d^
7	66.75 ± 0.73 ^d^	3.97 ± 0.06 ^d^	17.67 ± 0.37 ^c^
8	68.04 ± 0.11 ^e^	3.83 ± 0.10 ^cd^	18.03 ± 0.20 ^c^
9	66.68 ± 0.43 ^d^	3.67 ± 0.17 ^c^	17.84 ± 0.37 ^c^
10	50.46 ± 0.52 ^a^	8.92 ± 0.13 ^e^	8.41 ± 0.37 ^a^
11	50.41 ± 0.56 ^a^	9.19 ± 0.09 ^e^	8.40 ± 0.12 ^a^
12	50.73 ± 0.53 ^a^	9.59 ± 0.01 ^f^	8.49 ± 0.12 ^a^

Results represent average value (*n* = 6) ± standard deviation. Different letters in superscript of the same table column indicate the statistically significant difference between values at a level of significance of *p* < 0.05 (based on post hoc Tukey HSD test). L—brightness (black/white), a—greenness/redness, b—blueness/yellowness.

**Table 7:** Mineral characteristics of flake products from different types of corn.

Sample	N (%)	S (%)	B (mg/kg)	Ca (mg/kg)	Cu (mg/kg)	Fe (mg/kg)	K (mg/kg)	Mg (mg/kg)	Mn (mg/kg)	Na (mg/kg)	P (mg/kg)	Zn (mg/kg)
1	1.594 ± 0.01 ^ab^	0.455 ± 0.04 ^c^	7.305 ± 0.120 ^e^	506.2 ± 4.26 ^g^	10.72 ± 1.28 ^ab^	179.50 ± 15.84 ^e^	2237 ± 114 ^ab^	1001 ± 114.02 ^a^	11.25 ± 0.40 ^e^	35.92 ± 1.10 ^d^	2386 ± 256.59 ^a^	18.15 ± 1.16 ^a^
2	1.581 ± 0.0041 ^ab^	0.320 ± 0.039 ^ab^	6.236 ± 0.152 ^d^	423.3 ± 5.11 ^b^	15.15 ± 2.431 ^b^	149.70 ± 43.85 ^d^	2164 ± 93 ^a^	954.9 ± 53.06 ^a^	9.543 ± 0.97 ^d^	35.89 ± 0.77 ^d^	2408 ± 102.34 ^a^	20.95 ± 0.60 ^a^
3	1.523 ± 0.185 ^ab^	0.361 ± 0.068 ^abc^	5.805 ± 0.325 ^c^	247.1 ± 14.68 ^f^	38.99 ± 3.128 ^g^	80.63 ± 3.825 ^c^	2250 ± 267 ^ab^	965.8 ± 89.51 ^a^	8.16 ± 1.049 ^c^	35.68 ± 2.783 ^cd^	2578 ± 339.76 ^a^	35.48 ± 3.52 ^cd^
4	1.358 ± 0.162 ^a^	0.402 ± 0.073 ^bc^	6.182 ± 0.990 ^d^	202.4 ± 27.93 ^e^	19.02 ± 0.92 ^c^	78.56 ± 2.89 ^c^	2395 ± 187 ^abc^	869.6 ± 91.96 ^a^	6.955 ± 0.24 ^abc^	32.92 ± 2.40 ^abc^	2229 ± 274.84 ^a^	27.32 ± 2.05 ^c^
5	1.475 ± 0.226 ^ab^	0.371 ± 0.072 ^abc^	5.746 ± 0.383 b^c^	135.2 ± 1.62 ^d^	22.57 ± 0.945 ^d^	59.16 ± 59.16 ^bc^	2483 ± 243 ^abc^	886.9 ± 65.50 ^a^	6.507 ± 0.347 ^ab^	33.32 ± 1.12 ^abc^	2315 ± 90.95 ^a^	39.68 ± 0.80 ^d^
6	1.426 ± 0.293 ^ab^	0.255 ± 0.054 ^a^	5.935 ± 0.306 ^d^	113.9 ± 1.68 ^c^	32.68 ± 1.018 ^e^	46.42 ± 0.928 ^ab^	2519 ± 286 ^bc^	900.3 ± 35.36 ^a^	6.072 ± 0.63 ^a^	31.37 ± 0.71 ^a^	2381 ± 181.95 ^a^	20.37 ± 0.75 ^a^
7	1.59 ± 0.01 ^ab^	0.305 ± 0.070 ^ab^	4.920 ± 0.640 ^ab^	81.25 ± 0.75 ^ab^	6.64 ± 0.58 ^a^	35.93 ± 1.33 ^ab^	2398 ± 184 ^abc^	951.8 ± 120.69 ^a^	6.401 ± 0.64 ^a^	35.47 ± 0.70 ^d^	2408 ± 161.57 ^a^	24.00 ± 0.73 ^b^
8	1.564 ± 0.20 ^ab^	0.321 ± 0.050 ^ab^	5.691 ± 0.230 ^bc^	78.26 ± 0.61 ^a^	21.17 ± 0.45 ^cd^	39.71 ± 0.44 ^ab^	2443 ± 96 ^abc^	946.3 ± 50.40 ^a^	6.44 ± 0.42 ^ab^	35.77 ± 0.87 ^d^	2419 ± 372.88 ^a^	49.11 ± 1.53 ^e^
9	1.511 ± 0.16 ^ab^	0.351 ± 0.100 ^abc^	5.424 ± 0.860 ^bc^	114.10 ± 1.87 ^c^	7.65 ± 0.96 ^ab^	47.29 ± 0.66 ^b^	2301 ± 97 ^abc^	895.6 ± 8.13 ^a^	6.58 ± 0.51 ^ab^	32.89 ± 0.90 ^abc^	2268 ± 182.73 ^a^	20.6 ± 0.47 ^a^
10	1.697 ± 0.10 ^b^	0.280 ± 0.056 ^ab^	5.288 ± 0.102 ^abc^	97.00 ± 1.691 ^bc^	34.71 ± 1.496 ^ef^	36.02 ± 0.998 ^ab^	2514 ± 93 ^bc^	958.1 ± 57.122 ^a^	7.123 ± 0.640 ^abc^	35.61 ± 1.051 ^d^	2562 ± 127.653 ^a^	59.77 ± 1.38 ^f^
11	1.643 ± 0.103 ^ab^	0.356 ± 0.069 ^abc^	4.958 ± 0.498 ^ab^	86.22 ± 1.804 ^ab^	14.81 ± 0.950 ^b^	42.97 ± 1.213 ^ab^	2623 ± 130 ^c^	972.1 ± 31.331 ^a^	7.658 ± 0.837 ^bc^	31.9 ± 1.170 ^ab^	2643 ± 274.770 ^a^	33.76 ± 1.17 ^b^
12	1.671 ± 0.088 ^b^	0.347 ± 0.042 ^abc^	4.845 ± 0.129 ^a^	68.12 ± 1.26 ^a^	36.25 ± 0.709 ^fg^	30.32 ± 0.855 ^a^	2554 ± 122 ^bc^	911.4 ± 1.402 ^a^	7.122 ± 0.147 ^abc^	34.34 ± 0.896 ^bc^	2546 ± 171.825 ^a^	38.42 ± 1.10 ^cd^

Results represent average value (*n* = 6) ± standard deviation. Different letters in superscript in the same table column indicate the statistically significant difference between values, at a level of significance of *p* < 0.05 (based on post hoc Tukey HSD test).

**Table 8:** Biochemical properties of flake products from different types of corn.

	TPC (mg/g d.m)	TFC (mg/g d.m)	TAC (mg/g d.m)	DPPH-Test%	DPPH-Test ug and g ASCA
1	0.82 ± 0.13 ^a^	0.41 ± 0.04 ^a^	nd *	43.06 ± 1.01 ^ab^	2.10 ± 0.04 ^b^
2	0.85 ± 0.07 ^a^	0.42 ± 0.02 ^a^	nd	39.62 ± 1.08 ^a^	2.02 ± 0.01 ^a^
3	0.74 ± 0.03 ^a^	0.44 ± 0.06 ^ab^	nd	41.74 ± 1.02 ^ab^	2.07 ± 0.03 ^ab^
4	0.83 ± 0.07 ^a^	0.48 ± 0.04 ^bcd^	nd	45.41 ± 1.82 ^bc^	2.22 ± 0.04 ^c^
5	0.81 ± 0.19 ^a^	0.51 ± 0.04 ^cde^	nd	48.08 ± 1.08 ^c^	2.30 ± 0.17 ^c^
6	0.83 ± 0.05 ^a^	0.56 ± 0.03 ^de^	nd	49.97 ± 1.82 ^c^	2.30 ± 0.13 ^c^
7	1.16 ± 0.07 ^b^	0.57 ± 0.05 ^de^	0.02 ± 0.01 ^ab^	65.93 ± 1.82 ^de^	3.27 ± 0.04 ^d^
8	1.30 ± 0.04 ^b^	0.56 ± 0.01 ^ef^	0.04 ± 0.01 ^b^	70.34 ± 1.53 ^e^	3.61 ± 0.04 ^e^
9	1.33 ± 0.07 ^b^	0.61 ± 0.04 ^f^	0.04 ± 0.01 ^b^	71.71 ± 1.91 ^e^	3.70 ± 0.05 ^e^
10	2.54 ± 0.07 ^d^	0.74 ± 0.05 ^i^	0.44 ± 0.02 ^c^	93.88 ± 2.69 ^f^	7.01 ± 0.05 ^g^
11	2.35 ± 0.05 ^c^	0.83 ± 0.04 ^h^	0.37 ± 0.03 ^d^	96.25 ± 2.77 ^f^	7.33 ± 0.04 ^h^
12	2.37 ± 0.06 ^cd^	0.72 ± 0.03 ^g^	0.33 ± 0.03 ^e^	91.31 ± 2.55 ^f^	6.56 ± 0.04 ^f^

TPC—total phenolic content, TFC—total flavonoids content, TAC—total anthocyanins content; * nd = not detected. Results represent average value (*n* = 6) ± standard deviation. Different letters in superscript in the same table column indicate a statistically significant difference between values at a level of significance of *p* < 0.05 (based on post hoc Tukey HSD test).

**Table 9:** Z-score analysis of flake products from different types of corn.

Samples	Segment Z Score				Total Z Score
	S1	S2	S3	S4	
1	0.25	0.775919	0.782694	0.022228	0.39373
2	0.237484	0.775159	0.663111	0.021525	0.359754
3	0.370735	0.77582	0.554713	0.026851	0.374327
4	0.392256	0.939477	0.198479	0.072122	0.329855
5	0.497873	0.948249	0.294851	0.094881	0.393776
6	0.695851	0.937954	0.116665	0.126032	0.416424
7	0.45367	0.508628	0.403195	0.260882	0.391459
8	0.519961	0.531833	0.498063	0.30692	0.452355
9	0.71561	0.520823	0.21014	0.341012	0.447645
10	0.513748	0.021759	0.618253	0.938973	0.593643
11	0.552532	0.012602	0.373071	0.947071	0.545039
12	0.75	0.004676	0.396865	0.82111	0.571251

## Data Availability

Data are contained within the article.
